# Acute sprint performance responses to velocity-based versus traditional post activation performance enhancement interventions

**DOI:** 10.1371/journal.pone.0332479

**Published:** 2025-09-15

**Authors:** Murat Tutar, Sümeyye Genç, Atakan Çağlayan, Erkan Günay

**Affiliations:** 1 Faculty of Sports Sciences, İstanbul Gedik University, Turkey; 2 Faculty of Sports Sciences, Manisa Celal Bayar University, Turkey; Afyon Kocatepe University: Afyon Kocatepe Universitesi, TÜRKIYE

## Abstract

The aim of this study was to compare the acute effects of velocity-based training (VBT), traditional strength training (TSG) and a non- strength control condition (CG) on sprint performance in trained individuals. In Session 1, anthropometric measurements of the participants were taken, and then 1 repetition maximum (1RM) squat values were determined after explanation, visual demonstration and familiarization of the sprint test and VBT method. In the second session, all participants performed a sprint test for the control condition. In the third session, participants completed the post activation performance enhancement (PAPE) protocol specific to their group after a standard warm-up and then performed sprint tests. In the 0–10 m distance, only the group × time interaction was significant (p = .014); a performance decrease (p = .016) was observed in the TSG group. In the 0–20 m sprint, time (p < .001), group (p = .043), and interaction (p = .003) effects were significant, and a significant performance increase was found in the VBT and TSG groups (p < .001). In the 0–30 m sprint, group (p = .015) and interaction (p < .001) effects were significant; an improvement was observed in the VBT group (p < .001) and a decrease in the TSG group (p = .039). In conclusion, this study demonstrated that the VBT protocol produced a more noticeable acute improvement in sprint performance compared to TSG, even when applied with the same load absolute.

## Introduction

Acute strategies aimed at short-term enhancement of performance have become an important part of training planning, especially in sports that require high intensity and explosive strength, such as sprinting [1]. In this perspective, post-activation performance enhancement (PAPE), which has come to the spotlight in recent years, is characterized by transient performance enhancement following high-intensity or high-speed muscle activation [2]. PAPE is an effective method in sports that require explosive power, such as sprinting, and where speed and acceleration performance are critical [3,4], and various studies have shown that it improves sprint performance in the acute phase [5–7]. These effects are related to multiple physiological mechanisms, such as increased muscle temperature, increased motor unit activation, intramuscular fluid changes, and increased neuromuscular activity [8].

Many studies to demonstrate the PAPE effect have used traditional strength training protocols, which are usually performed with resistance and include controlled loading and tempo based on % of 1 RM [9,10]. However, these methods have limitations both in terms of responsiveness to individual differences and controllability of loading parameters [11–13]. For this reason, researchers have also conducted studies on velocity-based training (VBT), an approach where the load is regulated by bar velocity instead of absolute weight, as a potential method to optimize the PAPE effect [14–16].

VBT protocols provide real-time feedback based on individual bar velocity during exercise, allowing for both standardization of practice and more precise regulation of fatigue management and neuromuscular activation [17,18]. This approach is supported by recent findings confirming the validity and reliability of bar velocity measurement devices in resistance training and demonstrating that mean bar velocity recordings provide acceptable accuracy in predicting training loads [19].These factors suggest that VBT may be a useful approach in the context of PAPE, because high force production, controlled fatigue, and maintained movement speed may play an important role in the emergence of potentiation. In addition, it is believed that VBT provides a speed advantage in eccentric-concentric transitions and this may strengthen the PAPE effect by supporting physiological mechanisms such as motor unit synchronization [20].

Recent systematic reviews and meta-analyses have shown that speed-based strength training is effective on performance components such as sprinting compared to traditional 1%RM-based protocols [21–24]. These studies evaluated the application of VBT in the context of chronic training protocols, usually 6–10 weeks, and examined performance changes based on long-term adaptations. In addition, technological integrations based on real-time measurement of parameters such as bar velocity in studies and real field conditions have enabled a detailed evaluation of the physiological basis of VBT. However, the majority of these studies have not focused on the potentiation effects of VBT protocols in the acute phase. In addition, while most studies on VBT have focused on chronic training effects, they have only limitedly examined its potential on short-term performance outcomes. However, in the context of PAPE, particularly in skills requiring high speed such as sprinting, detecting acute potentiation effects is important for both improving training efficiency and optimizing pre-competition preparation strategies. Therefore, evaluating the acute effect mechanisms of VBT independently of chronic adaptations presents a complementary research area in the literature.

In this perspective, the aim of the aim of the study is to compare the acute effects of VBT, TSG, and CG interventions on sprint performance in trained individuals. We hypothesized that the VBT protocol would improve acute sprint performance compared to TSG and CG due to the advantages of real-time speed feedback and load management.

## Methods

### Experimental approach to the problem

**Fig 1:**
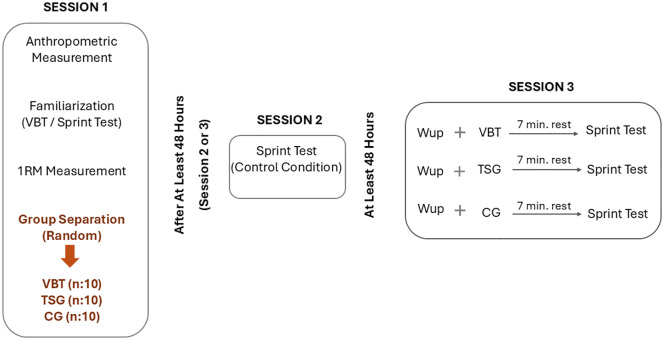
Experimental design. 1RM: One Repetition Maximum, VBT: Velocity-based Training, Wup: Warmup, TSG: Traditional Strength Group, CG: Control Group.

In this study, subjects participated in three experimental sessions. All interventions were conducted at the same time of the day (16:00–19:00 pm). In Session 1, anthropometric measurements of the participants were taken, then 1 repetition maximum (1RM) squat values were determined after explanation, visual demonstration and familiarization with the sprint test and VBT method. Participants were randomly assigned into three groups: VBT (n = 10), TSG (n = 10), and CG (n = 10) using Microsoft Excel’s random number generator. Subsequently, the order of the second and third sessions was randomized for each group using the same method to minimize order effects. In the second session, all participants performed a sprint test (assessed as a control condition). In the third session, participants performed a standard warm-up (5 minutes of jogging and dynamic mobilization exercises – 10–15 sec. each, 1 set; leg swings, walking lunges, hip circles) and completed the PAPE protocol specific to each group 3 minutes after. Then, after 7 minutes of passive rest [25], subjects performed sprint tests. A rest period of at least 48 hours was allowed between sessions to eliminate the fatigue effect (Fig 1). The recruitment period for participants was conducted between 05/12/2024 and 08/01/2025.

### Subjects

The study included 30 licensed male football players (age;18.10 ± 0.71 years, height;180 ± 5,7 cm, weight:73.23 ± 6.82 kg, training age:5.0 ± 1.0 years, 1RM;125 ± 20.87) who were actively football and strength training. Participants were selected from individuals who had previously adapted to the VBT method. The required sample size was calculated using G-Power software (version 3.1.9.4) with α = 0.05, power = 0.95 and effect size 0.43 based on a previous study [26]. Prior to the tests, participants were instructed to avoid intense physical activity for 72 hours and caffeine consumption for 24 hours. The study protocol was conducted in accordance with the latest version of the Declaration of Helsinki. Participants were informed about the potential risks and procedures of the study, and written informed consent was obtained. The study received ethical approval from İstanbul Gedik University Ethics Committee (354919).

### Anthropometric measurements

The height of the players was measured in cm with a Mesilife (MC-210, Türkiye) device. The body mass (kg) and body fat ratios of the players were measured on the Tanita (BC418, Japan) device, which measures the bioimpedance method, on the platform, barefoot, and wearing only shorts and t-shirts.

### 1 RM measurement

For the 1RM tests, Olympic bars (Eleiko, Sweden) and free weights (Gainzmach, Turkey) were used. Safety control of the depth of the squat distance is ensured by the safety bars of the power rack (Gainzmach, Turkey). According to the 1RM protocol of Pallarés et al. [27], each participant descended to the ~ 90° squat position with controlled eccentric contraction while standing in an upright position, and after waiting for 1.5 s with the contact of the Olympic bar on his shoulder with the safety bars of the power rack, he returned to the upright position by exhibiting concentric contraction at maximal speed. The test started with 10 min of warm-up with free jogging, dynamic stretches and calisthenic movements, followed by 10 reps of warm-up lifts with a 20 kg Olympic bar. Weights corresponding to an estimated 30% of 1RM were attached to the bar and a 1RM squat lift was performed. After 2 min of rest, weights between 100 kg and 20 kg were added and the squat one rep was continued. The test was continued until the participant was unable to lift the weight without assistance (usually 4–6 attempts) and the maximum weight he could successfully lift was recorded as 1RM.

### Bar velocity measurement

For the bar velocity measurement during the squat 12 repetitions of 1RM at 70%, a wireless velocity measurement device (VmaxPro VBT Tracker, Blaumann & Meyer Sports Technology, Magdeburg, Germany; accuracy ±0.02 m/s) with a three-axis accelerometer, gyroscope and magnetometer was used, the validity and reliability of which was established by Held et al. [28]. The device was calibrated before each lift and then connected to a tablet computer (iPad Pro, 11th inch (3rd generation), version 16.6.1; Apple, Inc.) via Bluetooth 5.0. After the sensor of the device was placed at 1/4 point of the bar, it was monitored and tracked along the squats with the Enode Pro application (version 2.0.5) compatible with the device and recorded at a sampling rate of 200 Hz. For the test to be accepted, the participant’s squat descending distance was set to a minimum of 30 cm. Participants performed each half-squat repetition at maximum velocity.

### PAPE interventions

#### Control Group (CG).

Participants in this group only performed the standard warm-up protocol (5 minutes of jogging and dynamic mobilization exercises – 10–15 seconds each, 1 set; Leg swings, Walking lunges, Hip circles) and were not included in any PAPE intervention afterwards..

#### Traditional Strength Group (TSG).

The Traditional Strength Group performed the PAPE protocol with 4 sets of 4 reps of squats using 1RM 40% resistance. Squats are performed at a tempo of 2-1-2. A rest period of 30 seconds was given between sets. All interventions were performed with free weights, at full squat depth and under the supervision of an experienced researcher while maintaining correct technical standards.

#### Velocity Based Training Group (VBT).

Velocity Based Training Group performed the PAPE protocol with 4 sets of 4 reps of squats using 1RM 40% resistance. The bar velocity during squats was set to be between 0.75–1.00 m/s^−1^. This velocity range was chosen because it is considered a strength-speed load [29]. Bar velocity was controlled by a VBT device monitored in real time. Repetitions that went out of the subjects target speed range were invalidated and repeated if necessary. A rest period of 30 seconds was given between sets. All interventions were performed with free weights, at full squat depth and under the supervision of an experienced researcher while maintaining correct technical standards.

### 30 meter sprint test

Sprint tests were measured using electronic timing gates (Witty Microgate, K_WIT001, Italy accuracy ±0.01 s) placed at the starting line (0 m) and at 10 m, 20 m and 30 m from the line. The gates were positioned 0.5 m above the ground. All athletes were started as soon as they feel ready in a standing position with their front foot 30 cm behind the start line. Standing starts were performed with the athlete’s preferred leg in front, which they regularly use during dynamic warmups before training on the field [30]. All sprint tests were conducted under consistent environmental conditions and on the same surface across all sessions. To ensure measurement standardization, participants wore the same pair of athletic shoes during each test session. Participants performed two maximal sprint trials with a 1-minute passive rest interval between each trial. The fastest sprint times obtained were used for statistical analysis.

### Statistical analysis

Statistical analyses were performed using SPSS (IBM SPSS Statistics 25). The normality of the data was assessed using the Shapiro-Wilk test, and the homogeneity of variances was evaluated with Levene’s test. A 3 (group: TSG, VBT, CG) × 2 (time: pre-test, post-test) mixed-design ANOVA was conducted to examine the main and interaction effects of group and time on 30 sec. sprint (0-10m, 0-20m and 0-30m). In cases of significant interactions, pairwise comparisons with Bonferroni adjustment were performed to analyze simple effects. Descriptive statistics (mean ± standard deviation) for each test were presented in a summary table. Additionally, absolute change (Δ) between pre- and post-test values was calculated for each group. To compare these Δ values between groups, a separate one-way ANOVA with Bonferroni post-hoc tests was performed. Effect sizes were also assessed with Cohen’s d [31]. Effect sizes were reported using partial eta squared (η²) for ANOVA and Cohen’s d for within-group changes; with thresholds for small, medium, and large effects defined as 0.01–0.06–0.14 (η²) and 0.2–0.5–0.8 (*d*), respectively [32].

## Results

**Fig 2:**
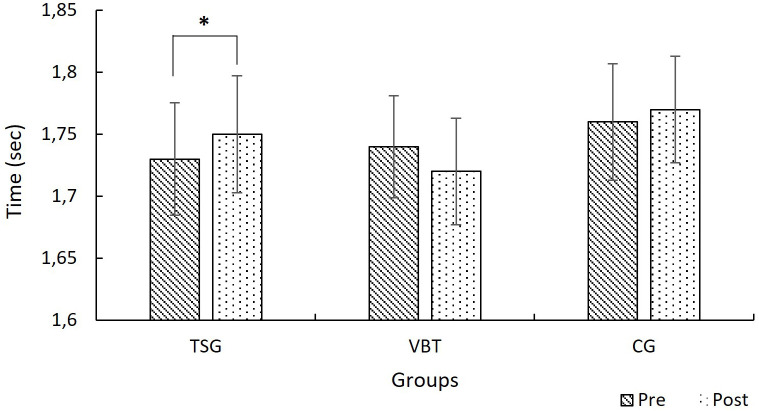
Mean 0-10 m sprint times for TSG, VBT, and CG groups at pre-test and post-test (with 95% CI). *p < 0.05.

In the mixed-design ANOVA, there was no significant main effect of time on 0–10-meter sprint performance (F(1, 27) = 0.49, p = .488, η² = .018) and no significant main effect of group (F(2, 27) = 0.57, p = .573, η² = .040). However, a significant time × group interaction was found (F(2, 27) = 4.99, p = .014, η² = .27). Simple effects analyses showed that the TSG group showed a significant decrease in performance.No statistically significant change was observed in the VBT group (p = .069), but slight improvement trend was observed compared to CG (p = .593) and TSG (Fig 2).

**Fig 3:**
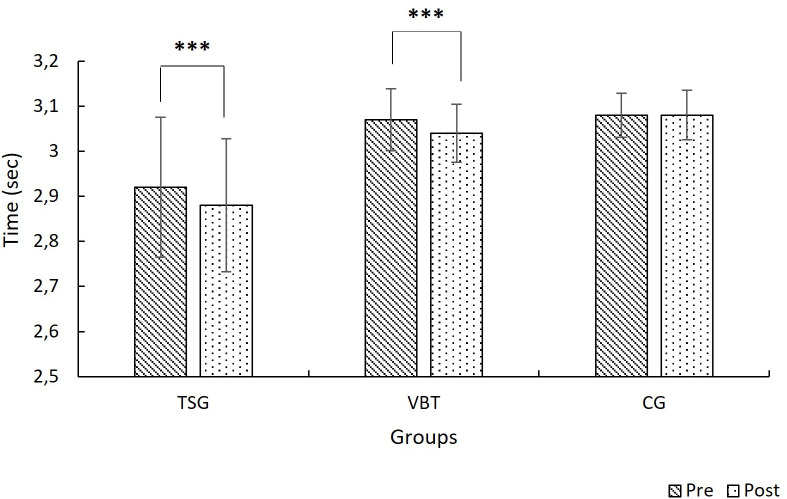
Mean 0-20 m sprint times for TSG, VBT, and CG groups at pre-test and post-test (with 95% CI). *** *p < *0.001.

The mixed ANOVA analysis of the 0–20-meter sprint performance showed a significant main effect of the time variable (F(1, 27) = 30.81, p < .001, η² = .53), indicating a significant improvement in performance from pre-test to post-test. Furthermore, the group main effect was also significant (F(2, 27) = 3.53, p = .043, η² = .20), indicating that there were performance differences between groups. The time × group interaction was also significant (F(2, 27) = 7.37, p = .003, η² = .35), indicating that changes over time differed between groups. According to the simple effects analysis, significant performance improvements were observed in the TSG and VBT groups (p < .001), indicating that performance increased significantly in both groups. In contrast, no significant change in sprint time was observed in the CG group (p = .867) (Fig 3).

**Fig 4:**
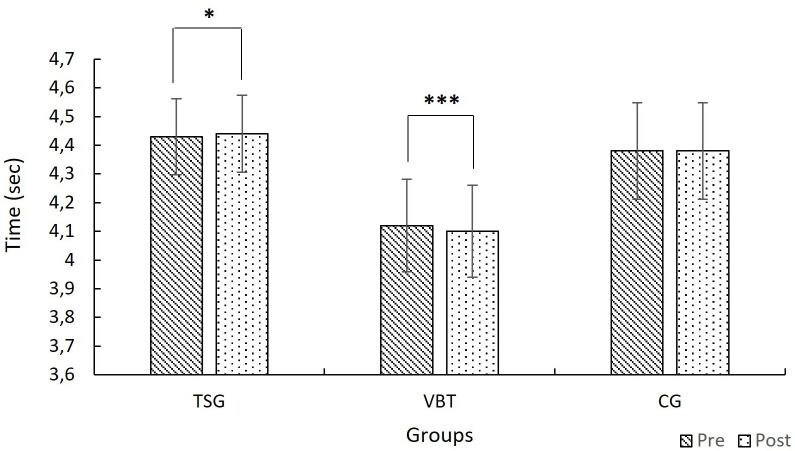
Mean 0-30 m sprint times for TSG, VBT, and CG groups at pre-test and post-test (with 95% CI). *p < 0.05; *** *p < *0.001.

Two-way mixed ANOVA results showed that the main effect of time was not significant, (F(1, 27) = 2.80, p = .106, η² = .094), whereas the main effect of group was statistically significant, (F(2, 27) = 4.91, p = .015, η² = .266). In addition, a significant interaction was found between time and group, (F(2, 27) = 10.66, p < .001, η² = .441). Comparisons showed a significant decrease in performance for the TSG group (p = .039). The VBT group showed a significant improvement in performance (p < .001), indicating an improvement in performance. The CG group did not show a significant change (p = .475) (Fig 4).

Group-based descriptive statistics for sprint tests are summarized in Table 1. The table shows the pretest and posttest means, standard deviations, delta values (Δ) and effect sizes (Cohen’s d) for each test. In the VBT group, negative Δ values indicating performance improvements all tests. In the TSG group, performance decrease were observed in the 0–10 m and 0–30 m sprints (Δ = +0.02 and +0.01). Although there was a small performance improvement in the 0−20 m sprint (Δ = −0.03), the effect size was small (*d* = −0.12). In CG, the change was very limited in all tests. This table supports the performance trends in line with the findings of the statistical analyses. ANOVA results for Δ values showed significant differences between groups: 0–10 m (F(2, 27) = 4.99, p = .014), 0–20 m (F(2, 27) = 7.37, p = .003), and 0–30 m (F(2, 27) = 10.66, p = .001). Bonferroni post-hoc analyses revealed that VBT achieved a significantly greater decrease in sprint time compared to TSG at 0–10 m (mean difference [MD] = –0.033 s, p = .012), indicating improved performance. In the 0–20 m, CG showed a significantly greater increase in sprint time compared to both VBT (MD = 0.023 s, p = .032) and TSG (MD = 0.031 s, p = .003), reflecting a decline in performance. In the 0–30 m, VBT demonstrated a significantly greater decrease in sprint time than both TSG (MD = –0.036 s, p = .001) and CG (MD = –0.020 s, p = .049), indicating improved performance.

**Table 1:** Group-based descriptive statistics and effect sizes.

Group	Test	Pre test (Mean ± SD)	Post test (Mean ± SD)	Δ	Cohen’s *d*
TSG (n = 10)	0-10 m	1.74 ± 0.08	1.76 ± 0.08	+0.02	0.28
	0-20 m	2.92 ± 0.25	2.89 ± 0.24	−0.03	−0.12
	0-30 m	4.44 ± 0.21	4.45 ± 0.22	+0.01	0.04
VBT (n = 10)	0-10 m	1.74 ± 0.07	1.73 ± 0.07	−0.01	−0.16
	0-20 m	3.07 ± 0.11	3.05 ± 0.10	−0.02	−0.18
	0-30 m	4.13 ± 0.26	4.10 ± 0.26	−0.03	−0.10
CG (n = 10)	0-10 m	1.77 ± 0.08	1.77 ± 0.07	+0.003	0.03
	0-20 m	3.08 ± 0.08	3.08 ± 0.08	0.000	0.00
	0-30 m	4.38 ± 0.27	4.38 ± 0.27	−0.003	−0.01

***Note.*** Values represent group means (± SD) for each test at pre- and post-test. Δ: Delta, Cohen’s *d:* Effect Size*,* TSG: Traditional Strength Group, VBT: Velocity-Based Training, CG: Control Group.

## Discussion

This study compared the acute effects of VBT and TSG protocols on sprint performance. The results showed that the VBT group achieved a significant performance increase especially in the 0–20 m and 0–30 m sprint distances. In the 0–10 m sprint, a positive trend approaching significance was observed in the VBT group (p = .069). The results show that the VBT protocol, which is based on an individualized loading model based on bar velocity, may have an enhancing effect on sprint performance in the acute period compared to the traditional protocol. These results, in this perspective, support the main hypothesis of the study.

In the literature, many studies examining the contribution of the PAPE effect on sprint performance have used protocols based on traditional 1%RM-based loading [33–37]. These protocols mostly include structured squat-like multi-joint exercises in the 85–95% 1RM band, and are assessed with sprint tests in the 10–30 m range [13,38,39]. For example, significant performance improvements have been reported in the 0–20 yard and 0–40 yard sprint distances following half squat exercises performed with 90% 1RM [40]. Similarly, it was reported that barbell hip thrust exercises performed with 50% and 85% 1RM load improved sprint performance in elite handball players by significantly decreasing 0–10 m and 0–15 m sprint times [41]. Furthermore, another study reported significant gains in 0–5 m, 0–10 m and 0–20 m sprint performance with the same exercise performed with 85% 1RM load in soccer players [42]. In tennis players, significant improvement in 0–5 m sprint time was observed after hip thrust protocol with 60% 1RM [34]. These results suggest that traditional PAPE protocols may be particularly effective on the first step (0–5 m), early acceleration (0–10 m) and acceleration phase (0–20 m) of sprint in different sports branches and at various loading intensities. Unlike these examples in the literature, our results investigated the acute effects on sprint performance with a low-intensity loading protocol such as 40% 1RM. The TSG results obtained showed that this loading pattern negatively affected the performance at distances 0–10 m (first step/acceleration phase) and 0–30 m (transition to maximum velocity/reach) of the sprint, and provided a significant improvement only at distances 0–20 m (mid-phase/acceleration). This may indicate that low-intensity loading fails to provide sufficient potentiation effect or limits the efficiency of transfer to the early and late phases of the sprint. The possible physiological mechanism behind this result is that explosive activities, such as sprinting, require powerful firing and synchronisation of motor units, as well as high-level involvement of type II muscle fibres. The 40% 1RM applied in TSG may not have been able to produce this critical stimulation, which is why it may not have improved performance, especially in the 0–10 m. In fact, the volume of the intervention may have triggered fatigue beyond stimulation in type I muscle fibres, which may have negatively affected performance in the 0–30 m.

VBT-based protocols provide an advantage in terms of fatigue control and individualization by determining the loading depending on the individual’s bar speed instead of a fixed 1%RM value [43]. Similar to this approach, recent studies have investigated the effect of PAPE with flywheel or bar speed matched protocols at different speeds and reported significant acute effects, especially on jump performance [44–46]. In our study, these approaches were adapted to a phased analysis of sprint performance and the effects of bar velocity-based squat loading were evaluated. In the literature, there are different results suggesting that VBT has positive effects on sprint performance in chronic adaptations [21]. In a 6-week study comparing traditional and VBT-based protocols, a group with a fixed 59–85% 1RM and a VBT group loaded according to bar velocity feedback were evaluated. According to the results, the VBT group showed more performance improvement in the 5 m (ES = −1.17), 10 m (ES = −0.93) and 20 m (ES = −1.27) sprint distances compared to the traditional approach; especially the first 10 m sprint time was found to be more effective [47]. Similarly, in a study comparing a 50–80% 1RM fixed load group with a VBT group adjusted with daily velocity feedback, improvements in 20 m sprint time were seen in both groups; however, the performance improvement was reported to be more apparent in the fixed load group (−1.99% vs. −0.95%) [48]. Furthermore, in another study comparing the VBT protocol configured in the velocity range of 00.75–1.00 m/s^−1^ with the traditional protocol using 40–60% 1RM constant load, both groups showed improvement in 10 m and 20 m sprint performance, but the difference between the groups was not significant [49]. These results show that although the literature suggests that VBT has the potential to improve sprint performance in the chronic phase compared to traditional, it is understood that the improvements obtained may vary depending on the application of the protocol (e.g., speed loss tolerance, rest period, load interval) and the measured sprint phase. The VBT protocol applied in our study shows that the results of the sprint tests performed after the squat exercise performed in the range of 00.75–1.00 m/s^−1^ show that a statistically significant performance increase was achieved especially in the 0–20 m and 0–30 m sprint distances, and there is a development trend close to significance in the 0–10 m distance. This may indicate that the current protocol may be more effective than the traditional one not only in the first step and acceleration phase (0–10 m), but also in the middle (10–20 m) and late (20–30 m) sprint phases. Since VBT is often associated with long-term training effects in the literature, this study provides evidence that VBT can improve sprint performance in the acute phase as well.

In conclusion, this study showed that the VBT protocol produced a more significant acute performance improvement in sprint performance compared to TSG, even when applied with the same absolute load. The focus on bar velocity makes VBT a more specific and effective priming tool for motor tasks that require high-speed force production, such as sprinting. The results support the preferred use of VBT protocols for acute performance enhancement in sports where explosive power and sprint capacity are critical. Moreover, thanks to its ability to be applied with lower loads, VBT has emerged as an athlete-friendly “priming” strategy that reduces the risk of injury.

### Limitations

This study has some limitations. First, the sample size is limited and consists only of athletes with specific training histories, which may limit the generalizability of the findings. Additionally, the neuro-muscular effects of the applied protocols were not directly assessed using methods such as electromyography. Therefore, the physiological mechanisms underlying the observed performance changes could only be interpreted indirectly. Variables such as rest periods, loading intensity, and speed zones may also have potentially influenced the results due to differences in individual responses. Furthermore, the fact that the VBT velocity range (0.75–1.00 m/s^−^^1^) was applied similarly in all participants is a limitation of the study.

### Future perspective

Future studies examining the effects of VBT protocols applied with different loading patterns (number of sets/reps, velocity zones, bar loading) and rest periods on performance will contribute to the development of more optimized training programs for trainers. In addition, comprehensive approaches that examine neuromuscular responses in biomechanical and electrophysiological aspects (e.g. sEMG, motor unit activation) will allow us to better understand the physiological mechanisms underlying VBT. Furthermore, future research should investigate the effects of individualizing VBT protocols on performance.

### Informed consent statement

Informed consent was obtained from all subjects involved in the study.

## References

[1] Hulet R, DeBeliso M, Lawrence MM. Effects of a sprint post-activation performance enhancement stimulus on discus throw performance in collegiate division i throwers: a pilot study. Int J Exerc Sci. 2025;18(3):251–62. doi: 10.70252/WHLI6641 40046508 PMC11881986

[2] Fischer J, Paternoster FK. Post-activation-performance enhancement: possible contributing factors. J Sports Sci Med. 2024;23(1):34–45. doi: 10.52082/jssm.2024.34 38455437 PMC10915613

[3] Gautam A, Singh P, Varghese V. Effects of postactivation potentiation enhacement on sprint and change-of-direction performance in athletes: a systematic review. J Bodyw Mov Ther. 2024;39:243–50. doi: 10.1016/j.jbmt.2024.02.006 38876634

[4] Brown TD, Vescovi JD, Vanheest JL. Assessment of linear sprinting performance: a theoretical paradigm. J Sports Sci Med. 2004;3(4):203–10. 24624004 PMC3938058

[5] Loturco I, Pereira LA, Moura TBMA, McGuigan MR, Boullosa D. Effects of different conditioning activities on the sprint performance of elite sprinters: a systematic review with meta-analysis. Int J Sports Physiol Perform. 2024;19(7):712–21. doi: 10.1123/ijspp.2024-0005 38823792

[6] Li L, Mo L, Liu Y, Mei T. The impact of different velocity losses on post-activation performance enhancement (PAPE) effects in sprint athletes: a pilot randomized controlled study. Sports (Basel). 2024;12(6):157. doi: 10.3390/sports12060157 38921851 PMC11207782

[7] Genç S. Post-activation performance enhancement (PAPE) interventions at different loads may enhance sprint performance in well-trained athletes. Spor Hekimliği Dergisi. 2024;59(3):088–93.

[8] Gallardo P, Giakas G, Sakkas GK, Tsaklis PV. Are surface electromyography parameters indicative of post-activation potentiation/post-activation performance enhancement, in terms of twitch potentiation and voluntary performance? A systematic review. J Funct Morphol Kinesiol. 2024;9(2):106. doi: 10.3390/jfmk9020106 38921642 PMC11205249

[9] Botton CE, Gama MCT, Oliveira CBT, de Oliveira FDA, Brusco CM. Post-activation performance enhancement, is this strategy recommended to increase the strength training volume? A systematic review. Sport Sci Health. 2024;21(1):15–25. doi: 10.1007/s11332-024-01260-2

[10] Krzysztofik M, Jarosz J, Urbański R, Aschenbrenner P, Stastny P. Effects of 6 weeks of complex training on athletic performance and post-activation performance enhancement effect magnitude in soccer players: a cross-sectional randomized study. Biol Sport. 2025;42(1):211–21. doi: 10.5114/biolsport.2025.139849 39758167 PMC11694194

[11] Chavda S, et al. Effect of post-activation potentiation on weightlifting performance and endocrinological responses. Appl Sci. 2025;15(2):748.

[12] Chiu LZF, Fry AC, Weiss LW, Schilling BK, Brown LE, Smith SL. Postactivation potentiation response in athletic and recreationally trained individuals. J Strength Cond Res. 2003;17(4):671–7. doi: 10.1519/1533-4287(2003)017<0671:ppriaa>2.0.co;2 14636093

[13] Seitz LB, de Villarreal ES, Haff GG. The temporal profile of postactivation potentiation is related to strength level. J Strength Cond Res. 2014;28(3):706–15. doi: 10.1519/JSC.0b013e3182a73ea3 23965945

[14] Krzysztofik M, Matykiewicz P, Celebanska D, Jarosz J, Gawel E, Zwierzchowska A. The acute post-activation performance enhancement of the bench press throw in disabled sitting volleyball athletes. Int J Environ Res Public Health. 2021;18(7):3818. doi: 10.3390/ijerph18073818 33917433 PMC8038688

[15] Tsoukos A, Brown LE, Terzis G, Veligekas P, Bogdanis GC. Potentiation of bench press throw performance using a heavy load and velocity-based repetition control. J Strength Cond Res. 2021;35(Suppl 2):S72–9. doi: 10.1519/JSC.0000000000003633 32398633

[16] Tsoukos A, Brown LE, Veligekas P, Terzis G, Bogdanis GC. Postactivation potentiation of bench press throw performance using velocity-based conditioning protocols with low and moderate loads. J Hum Kinet. 2019;68:81–98. doi: 10.2478/hukin-2019-0058 31531135 PMC6724597

[17] Banyard HG, Nosaka K, Haff GG. Reliability and validity of the load-velocity relationship to predict the 1RM back squat. J Strength Cond Res. 2017;31(7):1897–904. doi: 10.1519/JSC.0000000000001657 27669192

[18] Hernández-Belmonte A, Alegre LM, Courel-Ibáñez J. Velocity-based resistance training in soccer: practical applications and technical considerations. Strength Condition J. 2022;45(2):140–8. doi: 10.1519/ssc.0000000000000707

[19] Janicijevic D, Şentürk D, Akyildiz Z, Weakley J, García‐Ramos A. Using velocity recordings to predict squat repetitions to failure in high‐level wrestlers. Eur J Sport Sci. 2024;24(3):364–71. doi: 10.1002/ejsc.12094

[20] Cormie P, McGuigan MR, Newton RU. Developing maximal neuromuscular power: part 2 - training considerations for improving maximal power production. Sports Med. 2011;41(2):125–46. doi: 10.2165/11538500-000000000-00000 21244105

[21] Liao K-F, Wang X-X, Han M-Y, Li L-L, Nassis GP, Li Y-M. Effects of velocity based training vs. traditional 1RM percentage-based training on improving strength, jump, linear sprint and change of direction speed performance: a systematic review with meta-analysis. PLoS One. 2021;16(11):e0259790. doi: 10.1371/journal.pone.0259790 34793506 PMC8601436

[22] Orange ST, Hritz A, Pearson L, Jeffries O, Jones TW, Steele J. Comparison of the effects of velocity-based vs. traditional resistance training methods on adaptations in strength, power, and sprint speed: a systematic review, meta-analysis, and quality of evidence appraisal. J Sports Sci. 2022;40(11):1220–34. doi: 10.1080/02640414.2022.2059320 35380511

[23] Held S, Speer K, Rappelt L, Wicker P, Donath L. The effectiveness of traditional vs. velocity-based strength training on explosive and maximal strength performance: a network meta-analysis. Front Physiol. 2022;13:926972. doi: 10.3389/fphys.2022.926972 36035476 PMC9399433

[24] Włodarczyk M, Adamus P, Zieliński J, Kantanista A. Effects of velocity-based training on strength and power in elite athletes-a systematic review. Int J Environ Res Public Health. 2021;18(10):5257. doi: 10.3390/ijerph18105257 34069249 PMC8156188

[25] Wilson JM, Duncan NM, Marin PJ, Brown LE, Loenneke JP, Wilson SMC, et al. Meta-analysis of postactivation potentiation and power: effects of conditioning activity, volume, gender, rest periods, and training status. J Strength Cond Res. 2013;27(3):854–9. doi: 10.1519/JSC.0b013e31825c2bdb 22580978

[26] Dorrell HF, Smith MF, Gee TI. Comparison of velocity-based and traditional percentage-based loading methods on maximal strength and power adaptations. J Strength Cond Res. 2020;34(1):46–53. doi: 10.1519/JSC.0000000000003089 30946276

[27] Pallarés JG, Sánchez-Medina L, Pérez CE, De La Cruz-Sánchez E, Mora-Rodriguez R. Imposing a pause between the eccentric and concentric phases increases the reliability of isoinertial strength assessments. J Sports Sci. 2014;32(12):1165–75. doi: 10.1080/02640414.2014.889844 24575723

[28] Held S, Rappelt L, Deutsch J-P, Donath L. Valid and reliable barbell velocity estimation using an inertial measurement unit. Int J Environ Res Public Health. 2021;18(17):9170. doi: 10.3390/ijerph18179170 34501761 PMC8431394

[29] Signore N. Velocity-based training: How to apply science, technology, and data to maximize performance. In: Human Kinetics. 2021.

[30] Bellon CR, DeWeese BH, Sato K, Clark KP, Stone MH. Defining the early, mid, and late subsections of sprint acceleration in division I Men’s soccer players. J Strength Cond Res. 2019;33(4):1001–6. doi: 10.1519/JSC.0000000000003088 30789585

[31] Field A. Discovering statistics using IBM SPSS statistics. Sage Publications Limited; 2024.

[32] Goss-Sampson M. Statistical analysis in JASP: A guide for students. JASP; 2019.

[33] Sari C, Koz M, Salcman V, Gabrys T, Karayigit R. Effect of post-activation potentiation on sprint performance after combined electromyostimulation and back squats. Appl Sci. 2022;12(3):1481. doi: 10.3390/app12031481

[34] Fernández-Galván LM, Prieto-González P, Sánchez-Infante J, Jiménez-Reyes P, Casado A. The post-activation potentiation effects on sprinting abilities in junior tennis players. Int J Environ Res Public Health. 2022;19(4):2080. doi: 10.3390/ijerph19042080 35206269 PMC8871887

[35] Williams JJ, Herron RL, Spradley B, Saracino P. Postactivation potentiation effect of heavy sled towing on subsequent sprints. J Strength Cond Res. 2021;35(5):1229–33. doi: 10.1519/JSC.0000000000003863 33044362

[36] Chen X, Zhang W, He J, Li D, Xie H, Zhou Y, et al. Meta-analysis of the intermittent time of post-activation potentiation enhancement on sprint ability. J Sports Med Phys Fitness. 2023;63(1):86–94. doi: 10.23736/S0022-4707.22.13502-4 35620952

[37] Çabuk S, İnce İ. The acute effects of hip thrust and glute bridge exercises with different loads on sprint performance and horizontal force-velocity profile in adolescent soccer players: a post-activation performance enhancement approach. Eur J Sport Sci. 2025;25(2):e12255. doi: 10.1002/ejsc.12255 39832164 PMC11745155

[38] Chatzopoulos DE, Michailidis CJ, Giannakos AK, Alexiou KC, Patikas DA, Antonopoulos CB, et al. Postactivation potentiation effects after heavy resistance exercise on running speed. J Strength Cond Res. 2007;21(4):1278–81. doi: 10.1519/R-21276.1 18076255

[39] Bevan HR, Cunningham DJ, Tooley EP, Owen NJ, Cook CJ, Kilduff LP. Influence of postactivation potentiation on sprinting performance in professional rugby players. J Strength Cond Res. 2010;24(3):701–5. doi: 10.1519/JSC.0b013e3181c7b68a 20145565

[40] Atalağ O, Kurt C, Solyomvari E, Sands J, Cline C. Postactivation potentiation effects of back squat and barbell hip thrust exercise on vertical jump and sprinting performance. J Sports Med Phys Fitness. 2020;60(9):1223–30. doi: 10.23736/S0022-4707.20.10888-0 32586077

[41] Dello Iacono A, Padulo J, Seitz LD. Loaded hip thrust-based PAP protocol effects on acceleration and sprint performance of handball players. J Sports Sci. 2018;36(11):1269–76. doi: 10.1080/02640414.2017.1374657 28873044

[42] Dello Iacono A, Seitz LB. Hip thrust-based PAP effects on sprint performance of soccer players: heavy-loaded versus optimum-power development protocols. J Sports Sci. 2018;36(20):2375–82. doi: 10.1080/02640414.2018.1458400 29595081

[43] Guerriero A, Varalda C, Piacentini MF. The role of velocity based training in the strength periodization for modern athletes. J Funct Morphol Kinesiol. 2018;3(4):55. doi: 10.3390/jfmk3040055 33466983 PMC7739360

[44] Sun S, Yu Y, Niu Y, Ren M, Wang J, Zhang M. Post-activation performance enhancement of flywheel and traditional squats on vertical jump under individualized recovery time. Front Physiol. 2024;15:1443899. doi: 10.3389/fphys.2024.1443899 39403567 PMC11471904

[45] Yuan Z, Liao K, Zhang Y, Han M, Bishop C, Chen Z, et al. Optimal velocity loss threshold for inducing post activation potentiation in track and field athletes. Biol Sport. 2023;40(2):603–9. doi: 10.5114/biolsport.2023.119284 37077778 PMC10108751

[46] Wang X, Zhai H, Wei H. Acute effects of different intensities of flywheel half squat based on velocity on vertical jump performance in high-level athletes. Applied Sciences. 2025;15(8):4388. doi: 10.3390/app15084388

[47] Banyard HG, Tufano JJ, Weakley JJS, Wu S, Jukic I, Nosaka K. Superior changes in jump, sprint, and change-of-direction performance but not maximal strength following 6 weeks of velocity-based training compared with 1-repetition-maximum percentage-based training. Int J Sports Physiol Perform. 2021;16(2):232–42. doi: 10.1123/ijspp.2019-0999 32871553

[48] Jiménez-Reyes P, Castaño-Zambudio A, Cuadrado-Peñafiel V, González-Hernández JM, Capelo-Ramírez F, Martínez-Aranda LM, et al. Differences between adjusted vs. non-adjusted loads in velocity-based training: consequences for strength training control and programming. PeerJ. 2021;9:e10942. doi: 10.7717/peerj.10942 33828909 PMC7996068

[49] Arslanoğlu E, Arslanoğlu C, Çelgin GS, Bayram M, Mor A. The effect of velocity-based training on some performance parameters in football players. Inter J Disabilities Sports Health Sci. 2024;7(6):1256–64. doi: 10.33438/ijdshs.1536481

